# Supplementary material for: Phase-engineered synthesis of atomically thin te single crystals with high on-state currents
Source: Nat Commun. 2024 Feb 16;15:1435. doi: 10.1038/s41467-024-45940-6 (PMC10873424; doi:10.1038/s41467-024-45940-6)
Supplement: Supplementary file 1 — Supplementary Information [file 41467_2024_45940_MOESM1_ESM.pdf]

Supplementary Information:

Phase-Engineered Synthesis of Atomically Thin Te Single Crystals with High ON-State Currents

Jun Zhou[1,#], Guitao Zhang[1,#], Wenhui Wang[1,#], Qian Chen[1], Weiwei Zhao[1], Hongwei Liu[2], Bei Zhao[1,*], Zhenhua Ni[1,3,*], Junpeng Lu[1,3,*]

[1]*School of Physics and Key Laboratory of Quantum Materials and Devices of Ministry of Education, Southeast University, Nanjing 211189, China.*

[2]*Jiangsu Key Lab on Opto-Electronic Technology, School of Physics and Technology, Nanjing Normal University, 1 Wenyuan Road, Nanjing 210023, China.*

[3]*School of Electronic Science and Engineering, Southeast University, Nanjing 210096, China.*

[#]*These authors contributed equally: Jun Zhou, Guitao Zhang, and Wenhui Wang.*

[*]*e-mail: beizhao@seu.edu.cn; zhni@seu.edu.cn; phyljp@seu.edu.cn*

Table of contents

Supplementary Fig. 1 Geometry of Te single crystals.

Supplementary Fig. 2 Formation energies of Te atoms binding with the WS_2 substrate and the *β*-Te cluster at high flow rates.

Supplementary Fig. 3 Comparison of Te growth on different substrates.

Supplementary Fig. 4 Effect of the Ar gas flow rate on the growth process of *α*-Te nanosheets and *β*-Te nanoribbons.

Supplementary Fig. 5 Thickness characterization of synthesized *α*-Te nanosheets and *β*-Te nanoribbons.

Supplementary Fig. 6 Atomic structure of 2D Te crystals.

Supplementary Fig. 7 Effect of growth temperature on the growth process of *α*-Te nanosheets.

Supplementary Fig. 8 Effect of growth temperature on the growth process of *β*-Te nanoribbons.

Supplementary Fig. 9 Effect of growth time on the growth process of *α*-Te nanosheets.

Supplementary Fig. 10 Effect of growth time on the growth process of *β*-Te nanoribbons.

Supplementary Fig. 11 Band structures of monolayer/five-layer *α*-/*β*-Te crystals.

Supplementary Fig. 12 Calculated band structure and bandgap of *β*-Te.

Supplementary Fig. 13 Thickness-dependent electrical characterization of *α*-Te devices.

Supplementary Fig. 14 Electrical characterization of *β*-Te transistors.

Supplementary Fig. 15 XPS spectra of WS_2 and Te/WS_2.

Supplementary Fig. 16 Characterization of the optical and electrical properties of the monolayer WS_2 transistor.

Supplementary Fig. 17 The stability of Te single crystals.

Supplementary Fig. 18 Preparation of Te nanoribbons on large-area MoS_2.

Supplementary Table 1 The key device parameters of *β*-Te transistors.

Supplementary Table 2 Calculated carrier mobility and other relevant parameters along the armchair and zigzag directions for the *β*-Te monolayer at 300 K.

Supplementary Table 3 Comparison of the ON-current density and mobility with those of other 2D semiconductor FET transistors.

Supplementary References

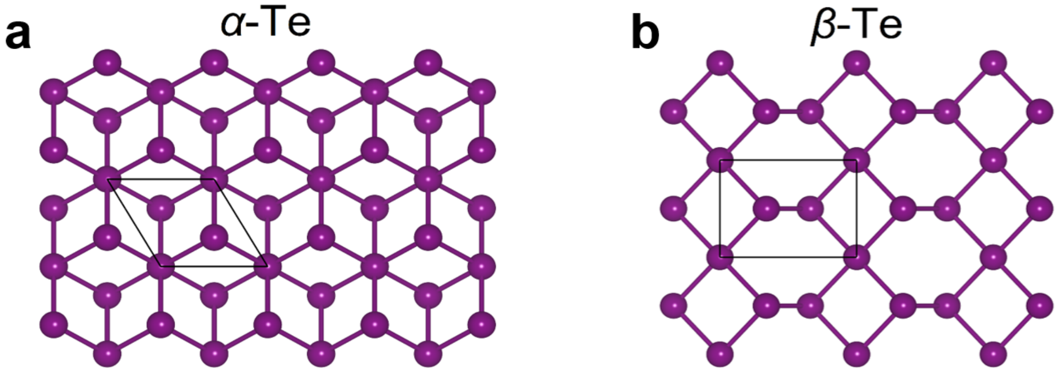

Supplementary Fig. 1 Geometry of Te single crystals. **a**, **b** Top view of the *α*-Te monolayer (**a**) and *β*-Te monolayer (**b**). The black box represents the unit cell. The purple balls denote Te atoms.

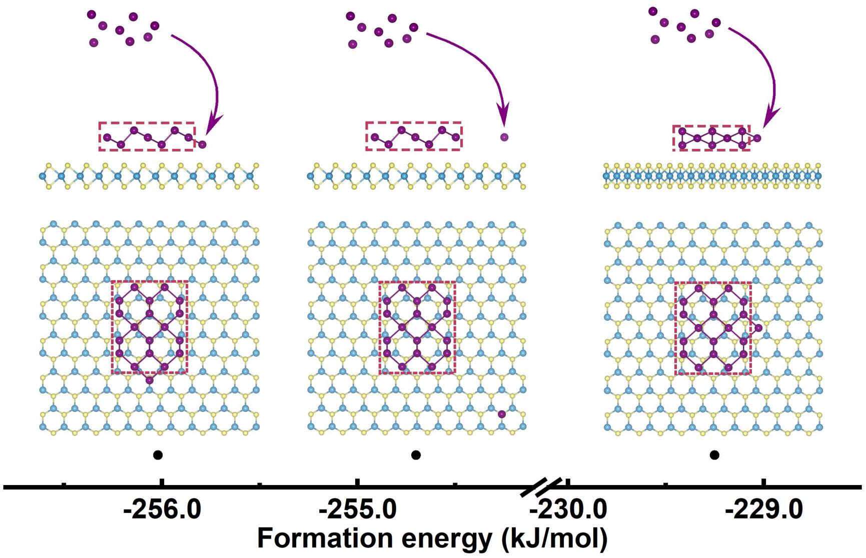

Supplementary Fig. 2 Formation energies of Te atoms binding with the WS_2 substrate and the *β*-Te cluster at high flow rates. Atom color code: Te, purple; W, blue; S, yellow. The insets are side views and top views of the atomic structure, and the red rectangular box represents the *β*-Te clusters.

To investigate the effect of interfacial interactions between Te atoms and the WS_2 substrate on the growth process, we calculated the formation energy E_f using formula (2) for two situations, including the case where Te atoms may be directly bound to the WS_2 substrate or extended to the Te clusters. As shown in Supplementary Fig. 2, the formation energy of Te directly binding to the WS_2 substrate is -254.7 kJ/mol, while the formation energies of Te adsorbed in the armchair and zigzag directions of the cluster are -256.0 and -229.2 kJ/mol, respectively. This further suggested that *β*-Te is more inclined to form nanoribbons along the armchair direction, which agrees well with our experimental results. Note that the growth of the individual Te atoms on the substrate and cluster is considered here, while in Fig. 1e, multiple Te atoms are considered. Therefore, the formation energy obtained here is higher than that in Fig. 1e, further indicating a stronger interaction between Te atoms.

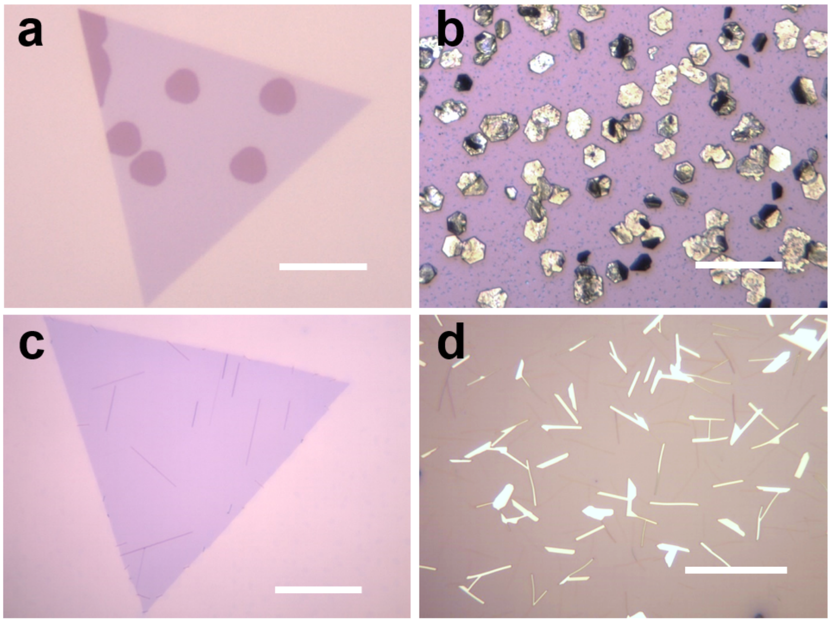

Supplementary Fig. 3 Comparison of Te growth on different substrates. a, **b** Optical microscopy images of the *α*-Te nanosheets on WS_2 (**a**) and SiO_2 (**b**). **c**, **d** Optical microscopy images of the *β*-Te nanoribbon on WS_2 (**c**) and SiO_2 (**d**), respectively. Scale bars: 10 μm.

As predicted by the theoretical calculations, the adjustment of the monolayer thickness is mainly due to the choice of substrate (Fig. 1f). Compared to SiO_2/Si, monolayer WS_2 (van der Waals substrate) is more favorable for the lateral growth of ultrathin Te crystals because of its atomic flatness, low surface energy and few defects under the same growth conditions (Supplementary Fig. 3). The thicknesses of *α*-Te and *β*-Te grown on SiO_2 can reach 100-500 nm (Supplementary Fig. 3b) and 50-200 nm (Supplementary Fig. 3d), while they are 0.4-17.6 nm and 0.4-14 nm on WS_2, respectively.

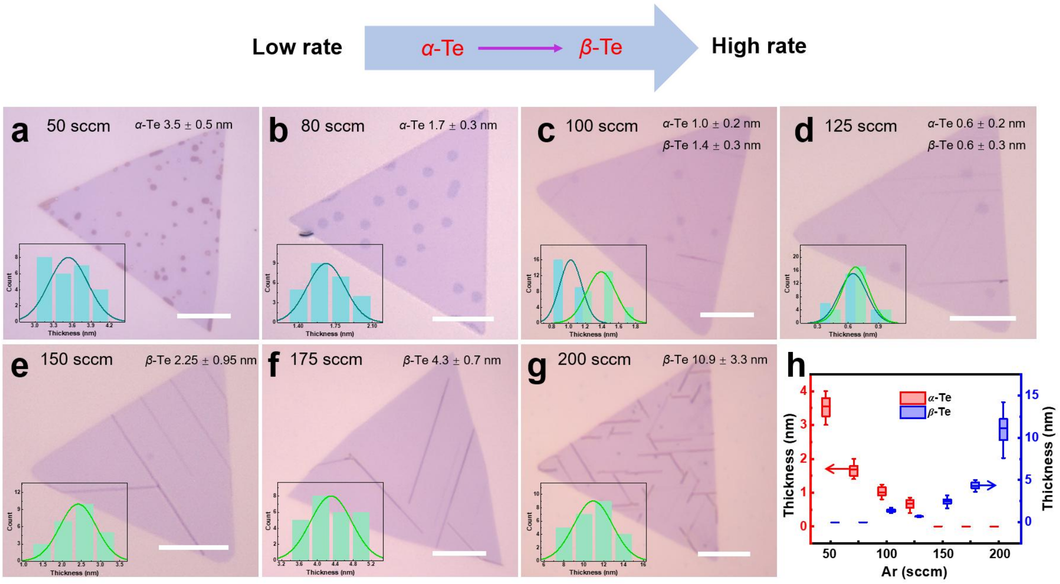

Supplementary Fig. 4 Effect of the Ar gas flow rate on the growth process of *α*-Te nanosheets and *β*-Te nanoribbons. a-g Optical microscopy images of tellurium growth on WS_2 with increasing Ar flow rate (50-200 sccm) at a growth temperature of 470 °C and growth time of 10 min. The insets in Supplementary Fig. 4**a-g** show the thickness statistics of the Te samples prepared at the corresponding gas flow rates and color codes for bar graphs: *α*-Te, blue; *β*-Te, green. **h** Thickness of *α*-Te nanosheets and *β*-Te nanoribbons as a function of the Ar flow rate. The error bars represent standard deviation, under different flow rates, 25 samples of α-Te and β-Te were collected for statistical analysis. Scale bars: 10 μm.

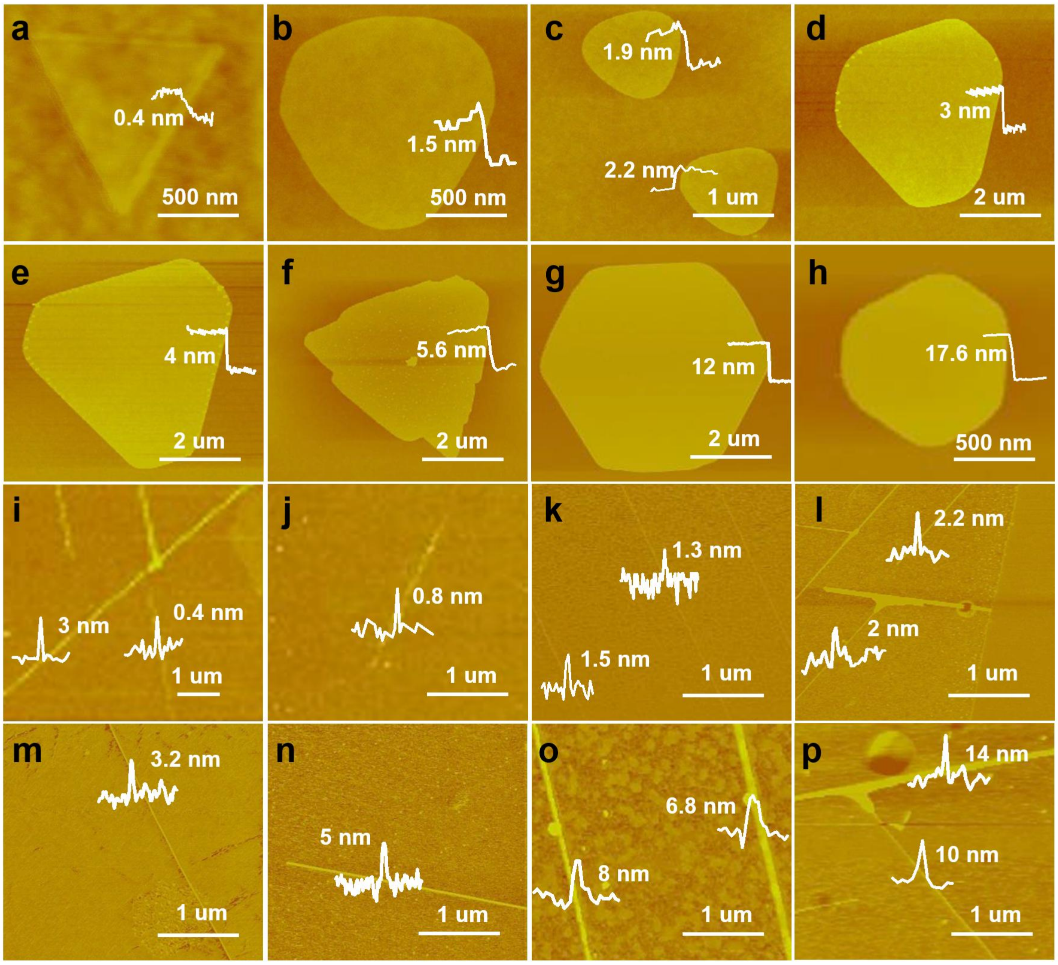

Supplementary Fig. 5 Thickness characterization of synthesized *α*-Te nanosheets and *β*-Te nanoribbons. a-**p** AFM images of the thickness variation of synthesized *α*-Te nanosheets (**a**-**h**) and *β*-Te nanoribbons (**i**-**p**).

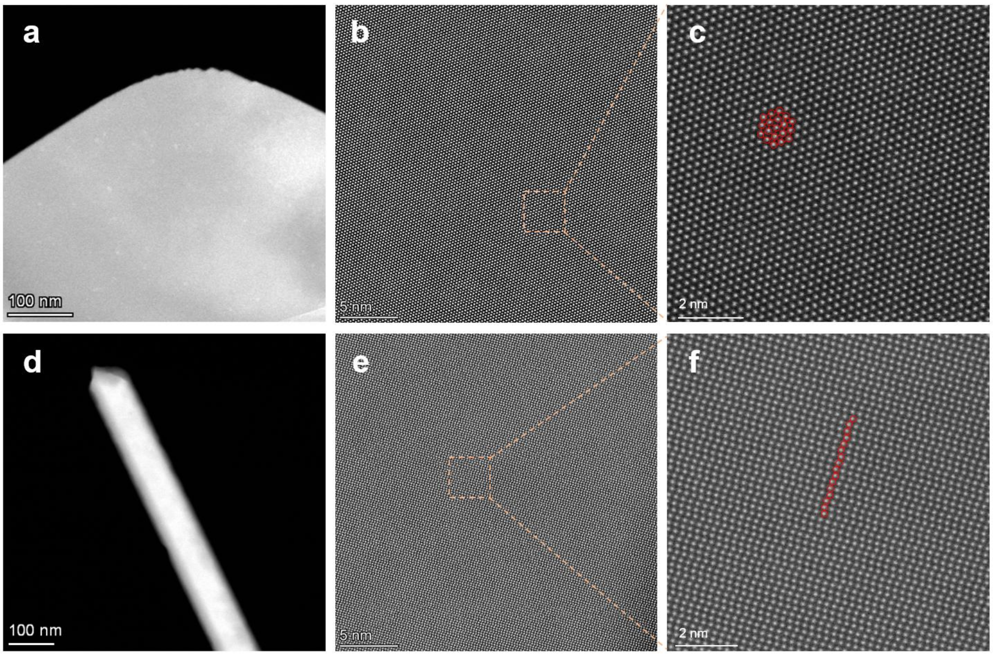

Supplementary Fig. 6 Atomic structure of 2D Te crystals. **a**, **d** Low-magnification transmission electron microscopy (TEM) images of *α*-Te nanosheets (**a**) and *β*-Te nanoribbons (**d**). **b**, **c**, **e**, **f** Atomic-resolution scanning transmission electron microscopy (STEM) images of *α*-Te nanosheets (**b**, **c**) and *β*-Te nanoribbons (**e**, **f**). **c** and **f** are STEM patterns taken near the orange dashed squares in **b** and **e**, corresponding to *α*-Te nanosheets and *β*-Te nanoribbons, respectively. The red circles in c and f represent Te atoms.

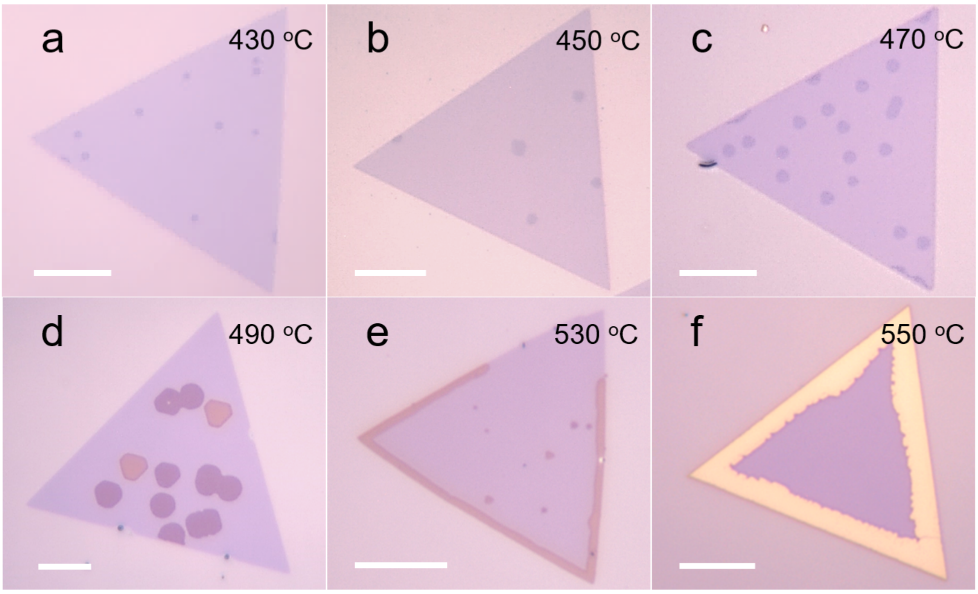

Supplementary Fig. 7 Effect of growth temperature on the growth process of *α*-Te nanosheets. a-f Optical microscopy images of *α*-Te nanosheets on WS_2 with increasing growth temperature (430-550 °C) at an Ar flow rate of 80 sccm and a growth time of 10 min. Scale bars: 10 μm.

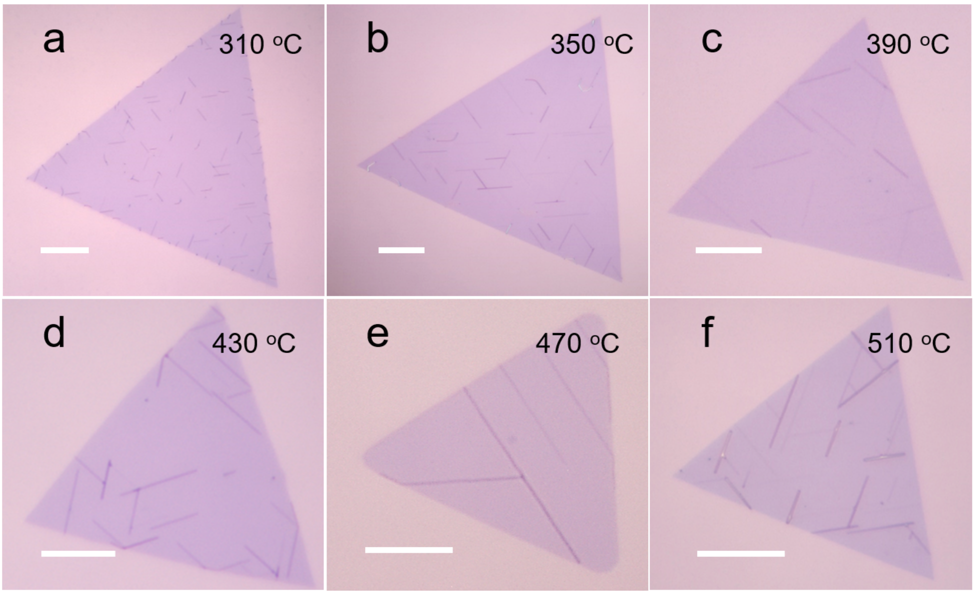

Supplementary Fig. 8 Effect of growth temperature on the growth process of *β*-Te nanoribbons. a-f Optical microscopy images of *β*-Te nanoribbon growth on WS_2 with increasing growth temperature (310-510 °C) at an Ar flow rate of 150 sccm and a growth time of 10 min. Scale bars: 10 μm.

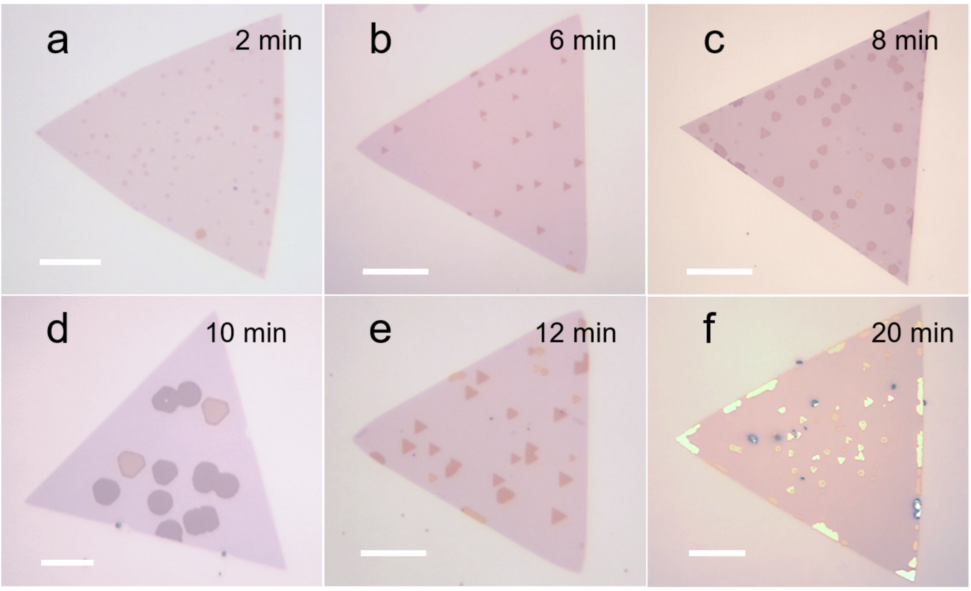

Supplementary Fig. 9 Effect of growth time on the growth process of *α*-Te nanosheets. a-**f** Optical microscopy images of *α*-Te nanosheets grown on WS_2 with increasing growth time (2-20 min) at an Ar flow rate of 80 sccm and a growth temperature of 490 °C. Scale bars: 10 μm.

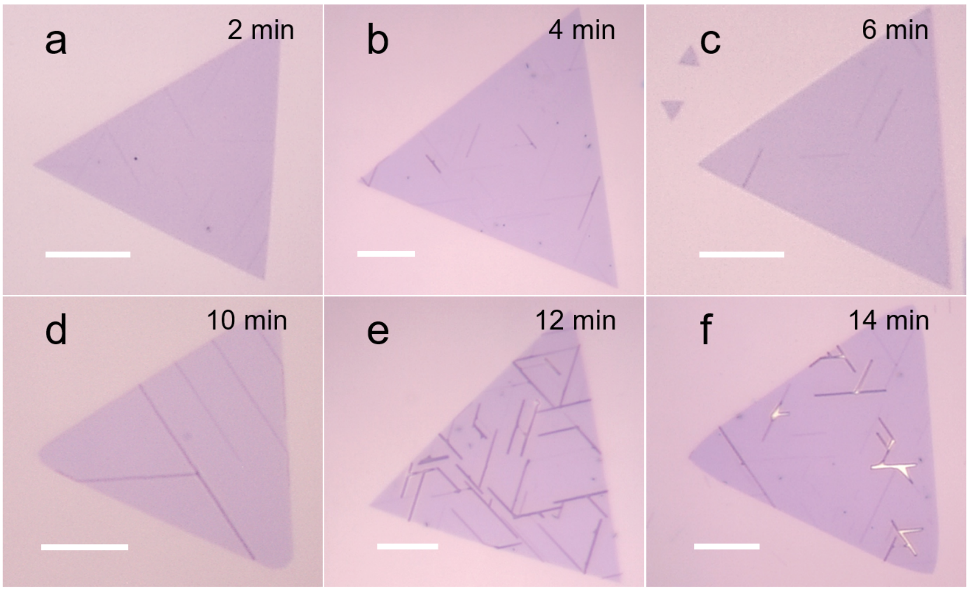

Supplementary Fig. 10 Effect of growth time on the growth process of *β*-Te nanoribbons. a-f Optical microscopy images of *β*-Te nanoribbon growth on WS_2 with increasing growth time (2-14 min) at an Ar flow rate of 150 sccm and a growth temperature of 470 °C. Scale bars: 10 μm.

With the same growth time of 10 min, Supplementary Fig. 7 and Supplementary Fig. 8 demonstrate the systematic evolution of the *α*-Te nanosheets and *β*-Te nanoribbons with increasing growth temperature at Ar flow rates of 80 sccm and 150 sccm, respectively. When the growth temperature was increased from 430 °C to 490 °C, hexagonal-shaped *α*-Te nanosheets were formed with thicknesses increasing from ~2 nm to ~4.6 nm and lateral average edge dimensions increasing from ~1.3 µm to ~5.2 µm (Supplementary Fig. 7a-d). When the growth temperature continued to increase from 530 °C to 550 °C, the *α*-Te nanosheets were concentrated along the edge of WS_2, and the thickness increased from ~9 nm to ~30 nm (Supplementary Fig. 7e-f). Similarly, as the growth temperature increased from 310 °C to 510 °C, the thickness of the *β*-Te nanoribbon increased from ~1.3 nm to ~5.6 nm, and the length increased from ~3 µm

to ~10 μm (Supplementary Fig. 8a-f). As the growth temperature increases, the growth behavior largely transforms from kinetically controlled to thermodynamically controlled, leading to thicker and larger samples. Supplementary Fig. 9 and Supplementary Fig. 10 demonstrate the effect of growth time on the α-Te nanosheets and β-Te nanoribbons at Ar flow rates of 80 sccm and 150 sccm and growth temperatures of 490 °C and 470 °C, respectively. When the growth time increases from 2 min to 10 min, the shape of most of the α-Te nanosheets transforms from triangular to truncated hexagonal as the thickness increases from ~1.5 nm to ~4.6 nm, and the lateral average edge increases from ~0.9 μm to ~5.2 μm (Supplementary Fig. 9a-d). When the growth time was increased to 12-20 min, the thickness of the α-Te nanosheets increased continuously to ~6.8-40 nm, but the shape became triangular, and the lateral average edge dimensions decreased to ~1.2-2 μm (Supplementary Fig. 9e-f). The thickness of the β-Te nanoribbons increases from ~1.2 nm to ~20 nm, and the length increases from ~6 μm to ~20 μm as the growth time increases from 2 to 14 min (Supplementary Fig. 10a-f). Prolongation of the growth time leads to a higher Te partial pressure and a greater supply of precursor, resulting in thicker and larger samples.

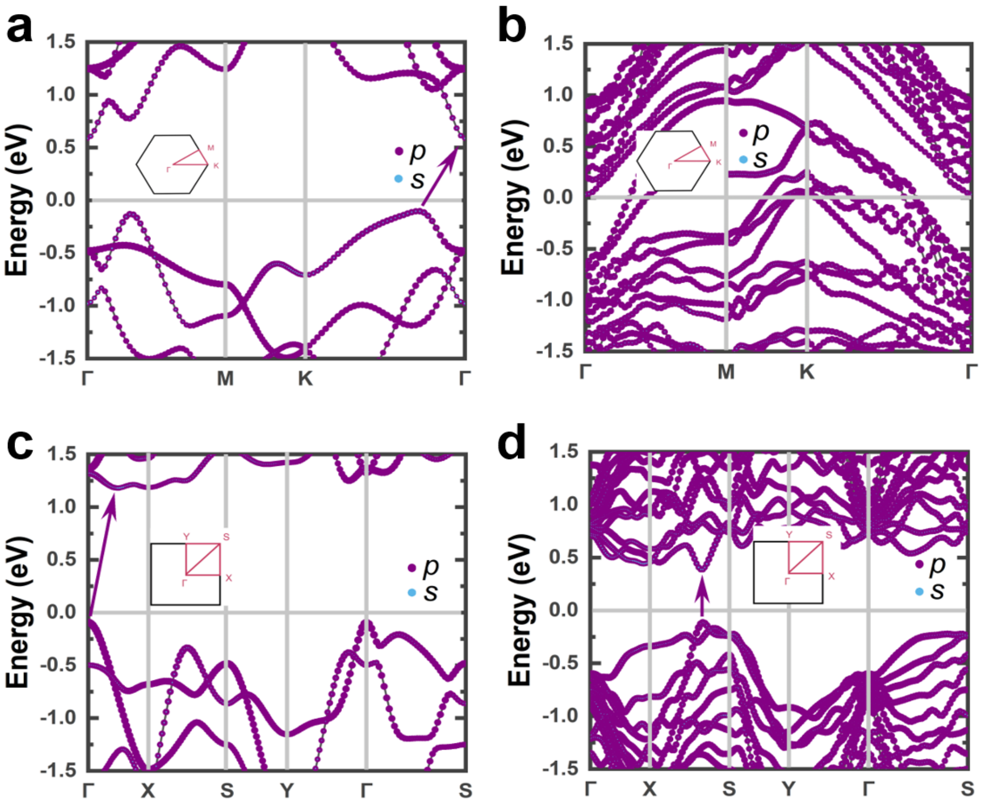

Supplementary Fig. 11 Band structures of monolayer/five-layer *α*-/*β*-Te crystals. a, **c** Band structures of a monolayer (~0.38 nm thick) of *α*-Te nanosheets (**a**) and *β*-Te nanoribbons (**c**). **b**, **d** Band structures of five layers (~2 nm thick) of *α*-Te nanosheets (**b**) and *β*-Te nanoribbons (**d**), respectively. The purple points and blue points indicate the projected weights of the *p* and *s* orbitals, respectively. The insets are Brillouin zones.

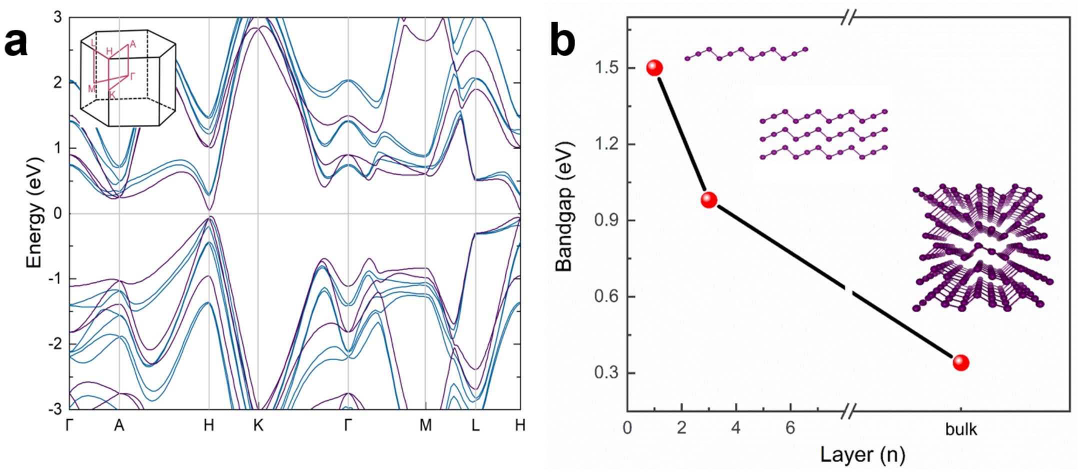

Supplementary Fig. 12 Calculated band structure and bandgap of *β*-Te. a Calculated band structure of bulk *β*-Te. The purple lines are calculated using the PBE functional, and the blue lines are calculated using the HSE functional with the SOC method. The inset is the Brillouin zone. **b** Bandgaps of *β*-Te nanoribbons as a function of the number of atomic layers determined via HSE+SOC functional calculations. The red points represent calculated bandgap values, and the purple balls represent Te atoms.

The bulk bandgap of *β*-Te obtained with HSE + SOC was approximately 0.34 eV (shown in Supplementary Fig. 12a). As shown in Supplementary Fig. 12b, we also performed HSE + SOC functional calculations on monolayer, trilayer and bulk *β*-Te as an example for bandgap determination. As the number of layers decreased, the band gap of *β*-Te gradually increased, which was consistent with the results of the PBE calculations. An increase in the number of atoms in multilayer structures leads to a substantial escalation in computational demands. Due to the intricacies of electronic interactions, this escalation is not linear. Considering the burden of calculation and the similarity of band structures between PBE and HSE + SOC, we used only the PBE functional in our work to investigate the influence of material thickness on the band structure of Te.

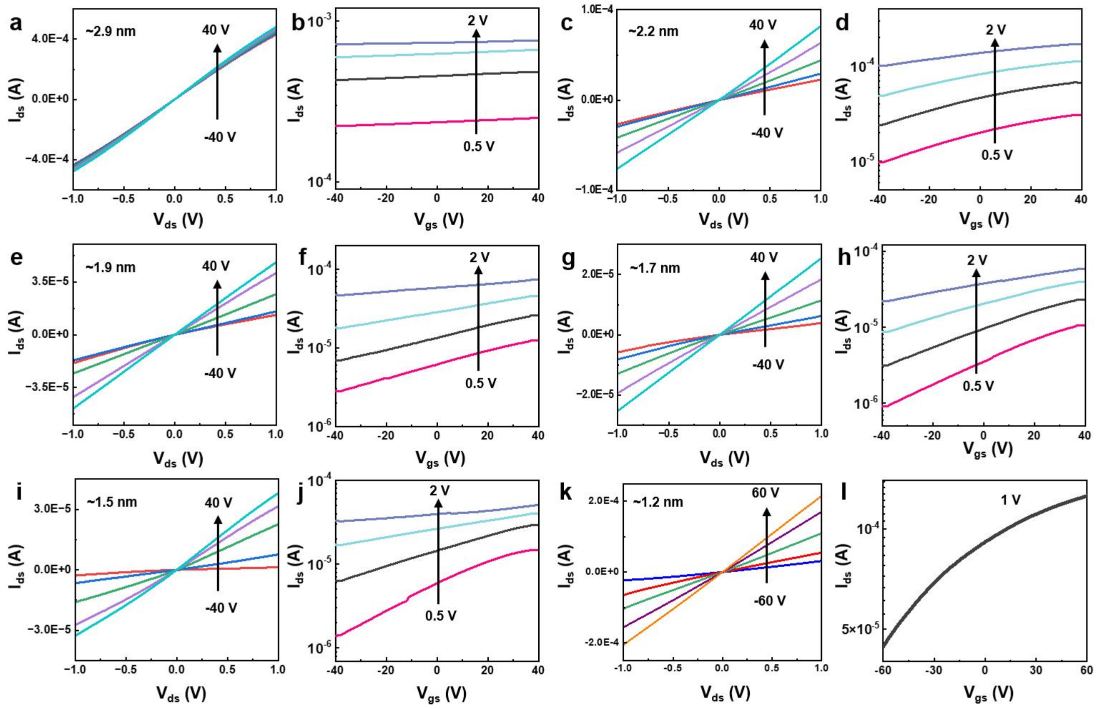

Supplementary Fig. 13 Thickness-dependent electrical characterization of *α*-Te devices. **a**, **c**, **e**, **g**, **i** Output curves under various gate voltages from -40 V to 40 V (20 V step) and **k** Output curves under various gate voltages from -60 V to 60 V (30 V step) of different *α*-Te/WS_2 devices with thicknesses in the range of 1.2~2.9 nm. **b**, **d**, **f**, **h**, **j**, **l** The transfer curves under various bias voltages (red, 0.5 V; black, 1 V; blue, 1.5 V; bluish violet, 2 V) of different *α*-Te/WS_2 devices with thicknesses in the range of 1.2~2.9 nm.

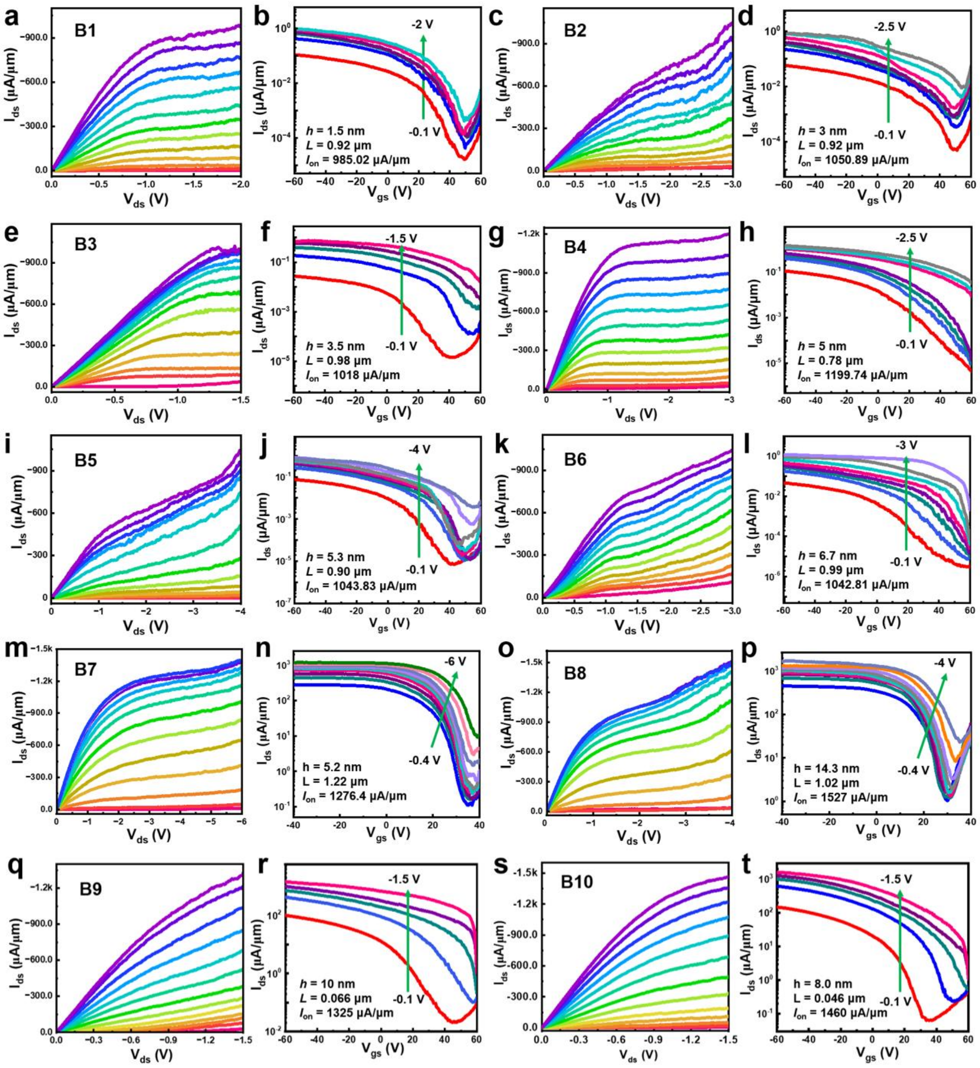

Supplementary Fig. 14 Electrical characterization of *β*-Te transistors. a-**l** Output (**a**, **c**, **e**, **g**, **i**, **k**) and transfer (**b**, **d**, **f**, **h**, **j**, **l**) curves of *β*-Te transistors of different thicknesses on WS_2/SiO_2 substrates. **m**-**p** Output (**m**, **o**) and transfer (**n**, **p**) curves of *β*-Te nanoribbon FETs on h-BN/80 nm thick Si_3N_4/Si substrates with thicknesses of 5.2 nm and 14.3 nm, respectively. **q**-**t** Output (**q**, **s**) and transfer (**r**, **t**) curves of the *β*-Te devices on h-BN/80-nm Si_3N_4/Si substrates with channel lengths of ~66 nm and ~46 nm, respectively. In the Output curves of *β*-Te transistors: in B1-B6 and B9-B10, the various gate voltages from 60 V to -60 V (-10 V step); and in B7-B8, the various gate voltages from 40 V to -40 V (-5 V step). In the transfer curves of *β*-Te transistors: in B1- B10, the various gate voltages from -0.1 V to -6 V correspond to different colored lines: red, -0.1 V; blue, -0.4 V; dark blue, -0.7 V; purple, -1 V; pink, -1.5 V; azure blue, -2 V; gray, -2.5 V; light purple, -3 V; orange, -3.5 V; blue-gray, -4 V; light pink, -5 V; olive-green, -6 V.

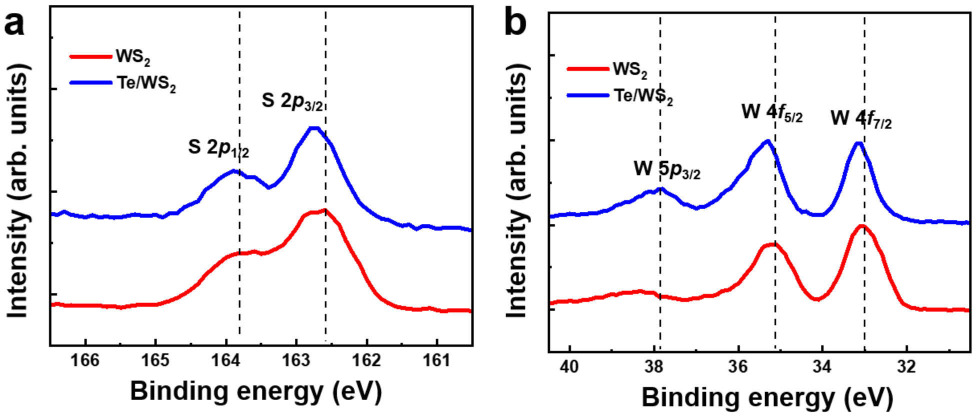

Supplementary Fig. 15 XPS spectra of WS_2 and Te/WS_2. a, b) XPS spectra of the S 2*p* (**a**) and W 4*f* (**b**) core-level regions for the WS_2 and Te/WS_2 heterostructures. The variation in the W $4f_{5/2}$/W $4f_{7/2}$ ratio is attributed to the oxidation of WS_2 itself due to prolonged exposure of NaCl-assisted WS_2 to air.

In addition, the XPS spectra of WS_2 and the β-Te/WS_2 heterostructure reveal the role of WS_2 in device performance (Supplementary Fig. 15). In the XPS spectra of WS_2, the peaks at 162.6 and 163.8 eV correspond to the S $2p_{3/2}$ and S $2p_{1/2}$ orbitals of divalent sulfide ions, respectively. The W peaks detected at 33.0, 35.1, and 38.0 eV correspond to W $4f_{7/2}$, W $4f_{5/2}$, and W $5p_{3/2}$, respectively. Both the W 3*d* and S 2*p* peaks blueshift (~0.2 eV) in the β-Te/WS_2 heterostructure, indicating very slight electron transfer from Te to WS_2. Moreover, the p-type characteristics of β-Te are enhanced, thereby enhancing the electrical properties of β-Te semiconductor devices.

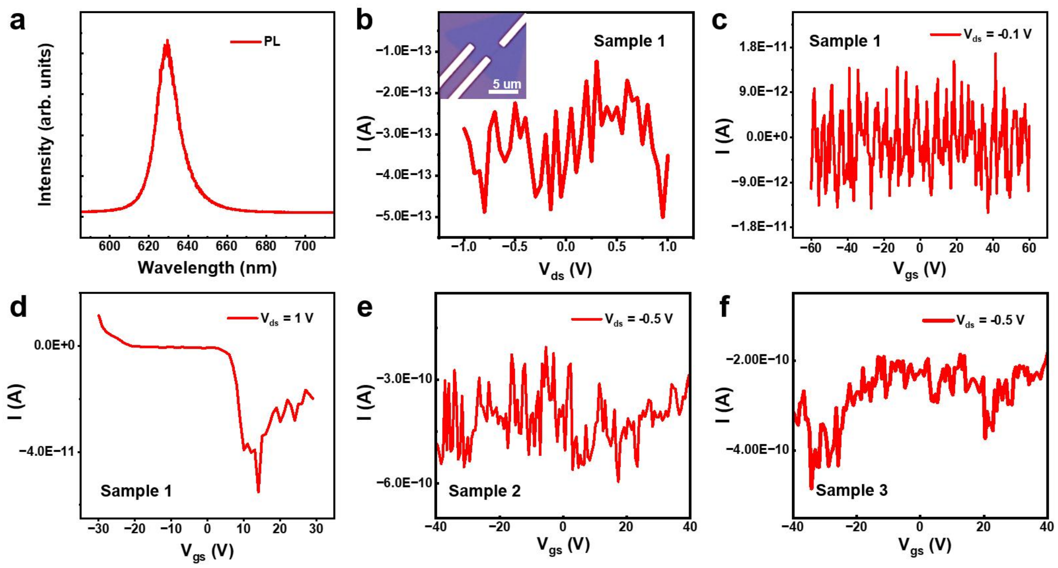

Supplementary Fig. 16 Characterization of the optical and electrical properties of the monolayer WS_2 transistor. **a** Photoluminescence image of monolayer WS_2. **b** Output curves of the monolayer WS_2 transistor numbered Sample 1. **c**, **d** Transfer curves of the monolayer WS_2 transistor numbered Sample 1 at V_{ds} = -0.1 V and V_{ds} = 1 V, respectively. **e**, **f** Transfer curves of Sample 2 (**e**) and Sample 3 (**f**) at V_{ds} = -0.5 V.

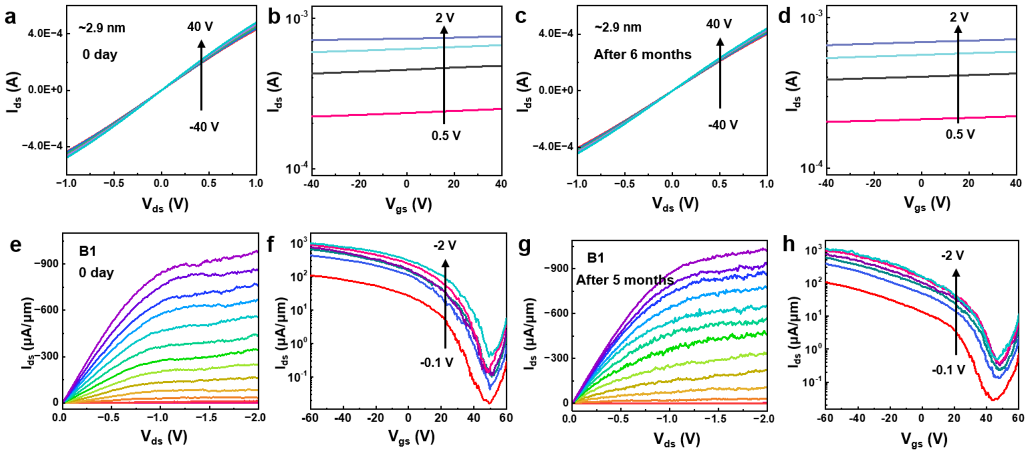

Supplementary Fig. 17 The stability of Te single crystals. a-d Output curves under various gate voltages from -40 V to 40 V (20 V step) and transfer curves under various bias voltages from 0.5 to 2 V (0.5 V step) for the α-Te device at 0 (**a**, **b**) and 6 months (**c**, **d**). **e-h** Output curves under various gate voltages from 60 V to -60 V (-10 V step) and transfer curves under various bias voltages from -0.1 (red) to -1 V (purple) (-0.3 V step) and from -1 V (purple) to -2 (azure blue) (-0.5 V step) for the β-Te transistor at 0 (**e**, **f**) and 5 months (**g**, **h**), respectively.

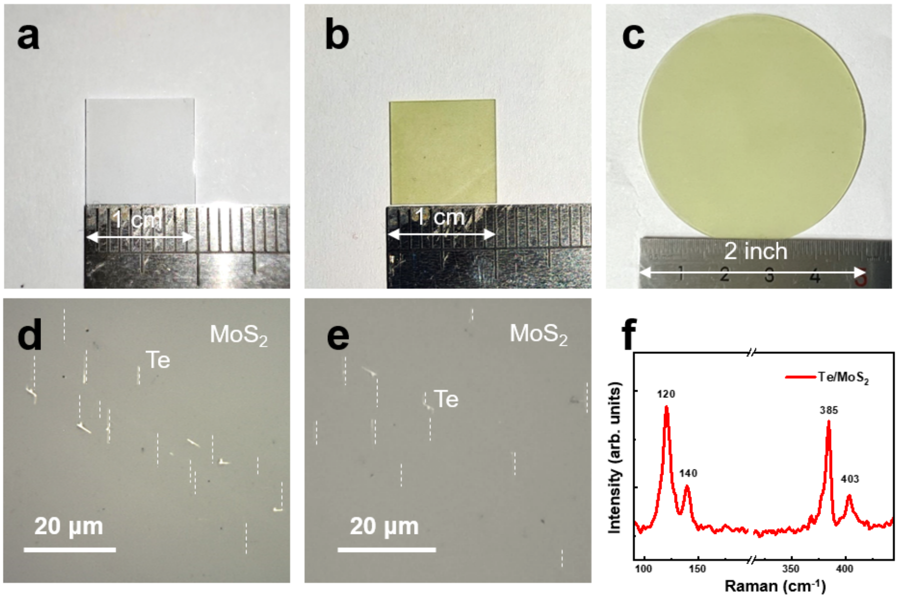

Supplementary Fig. 18 Preparation of Te nanoribbons on large-area MoS_2. a Sapphire substrate. **b**, **c** Optical image of 1×1 cm^2 (**b**) and 2-inch (**c**) MoS_2 grown on sapphires. **d-f** Optical images (**d**, **e**) and Raman spectrum (**f**) of *β*-Te nanoribbons growing on MoS_2.

Supplementary Table 1 The key device parameters of *β*-Te transistors.

Sample	Substrate	V_{ds} (V)	L (μm)	W (nm)	Thickness (nm)	Current density (μA μm^{-1})
B1	WS_2/SiO_2	-2	0.92	16.2	1.5	985.02
B2	WS_2/SiO_2	-3	0.92	27	3	1050.89
B3	WS_2/SiO_2	-1.5	0.98	33	3.5	1018
B4	WS_2/SiO_2	-3	0.78	77	5	1199.74
B5	WS_2/SiO_2	-4	0.90	47	5.3	1043.83
B6	WS_2/SiO_2	-3	0.99	49.6	6.7	1042.81
B7	h-BN/Si_3N_4	-6	1.22	29.3	5.2	1276.4
B8	h-BN/Si_3N_4	-4	1.02	36	14.3	1527
B9	h-BN/Si_3N_4	-1.5	0.066	44	10.0	1325
B10	h-BN/Si_3N_4	-1/-1.5	0.046	35.6	8.0	1270/1460

Supplementary Table 2 Calculated carrier mobility and other relevant parameters along the armchair and zigzag directions for the *β*-Te monolayer at 300 K.

		C (N/m)	*m** (m_0)	E_1 (eV)	*μ* ($cm^2\ V^{-1}\ s^{-1}$)
Electron	Armchair	14.47	0.97	0.20	81.22
	Zigzag	26.15	0.18	-7.98	219.33
Hole	Armchair	14.47	0.28	0.71	1134.16
	Zigzag	26.15	0.11	-6.56	1346.58

To further explore the electrical properties, we also calculate the carrier mobility of *β*-Te based on the deformation potential theory and the effective mass approximation, which is expressed by the following formulas for a 2D system[1]:

$$\mu_x = \frac{e\hbar^3(5C_{11}+3C_{22})/8}{k_B T(m_x)^{3/2}(m_y)^{1/2}(9E_{1x}^{\ 2}+7E_{1x}E_{1y}+4E_{1y}^{\ 2})/20} \tag{1}$$

$$\mu_y = \frac{e\hbar^3(3C_{11}+5C_{22})/8}{k_B T(m_x)^{1/2}(m_y)^{3/2}(4E_{1x}^{\ 2}+7E_{1x}E_{1y}+9E_{1y}^{\ 2})/20} \tag{2}$$

where C_{11} and C_{22} are the elastic stiffness constants, m_x and m_y are the effective masses, and E_{1x} and E_{1y} are the deformation potential constants along two different directions. The calculated hole mobilities are as high as 1134 (armchair) and 1346 $cm^2\ V^{-1}\ s^{-1}$ (zigzag), which are much greater than those of most common two-dimensional materials such as MoS_2 and WS_2[2,3]. The extremely high hole mobility of *β*-Te leads to good conductivity and a very high ON current.

Supplementary Table 3 Comparison of the ON-current density and mobility with those of 2D semiconductor transistors as supplementary information to Figure 5f.

	I_{ON} (μA/μm)	μ_{FE} (cm^2 V^{-1} s^{-1})	t (nm)	L_{CH}	V_{ds} (V)	Ref. in manuscript
Te	116	/	12.3	6.8 μm	1	2
	/	450	12	/	/	
Te	1060	/	11.1	300 nm	1.4	8
	/	700	16	/	/	
WSe_2	900	/	1.2	1.8 μm	0.1	38
	/	137	/	/	/	
WSe_2	2.68	12.1	1.6	1 μm	1.5	39
$PtSe_2$	4	/	8	1.5 μm	0.5	40
	/	210	11	2.95 μm	/	
$ZrSe_2$	20	/	~3.6	320 nm	2.5	41
	/	1.5	~3.6	530 nm	1	
$HfSe_2$	30	/	~4.8	140 nm	2.5	41
	/	4	~3.6	475 nm	1	
Se	20	/	16	4 μm	3	42
	/	0.26	/	/	/	
WS_2	350	/	0.65	120 nm	1.5	43
	/	20	0.65	26 nm	/	
ZnO	32.5	/	~17	1.5 μm	/	44
	/	~179	/	/	/	
As	1.3	51	0.7	2 μm	2	45
MoS_2	1270	/	1.4	50 nm	2.5	28
	/	122.6	1.4	8 μm	/	
MoS_2	450	/	0.65	500 nm	1	29
	/	102.6	0.65	/	/	
MoS_2	/	55	0.65	/	/	43
	1135	/	0.65	35 nm	/	
MoS_2	830	104	3.8	82 nm	2	46
MoS_2	470	/	0.65	~82 nm	/	47
	/	24.6	/	100 nm	1	
MoS_2	400	556	1.95	/	/	48
MoS_2	600	115	0.65	/	/	49
$MoTe_2$	112	/	0.7	4 μm	20	50
	/	32	/	/	/	
PtS_2	8.5	/	6.2	6.7 μm	0.9	51
	/	62.5	10	/	/	
SnO_2	~1.5	/	/	3.8 μm	/	52
	/	~100	~55	4.95 μm	1	

GeAs	~0.2	/	/	~1 μm	0.1	53
	/	100	~ 10	/	/	
SnSe	300	254	10	~2 μm	1	54
b-P	/	~1000	10	/	/	55
	~1.1	/	5	4.5 μm	2	
$MoSe_2$	15.6	121	150	13.1 μm	10	56
Te	1527	226.9	14.3	1.02 μm	4	This work
	/	690.7	12	5.90 μm	2.5	

Supplementary References

1. Lang, H. F. et al. Mobility anisotropy of two-dimensional semiconductors. *Phys. Rev. B* **94**, 235306 (2016).
2. Cai, Y. Q. et al. Polarity-reversed robust carrier mobility in monolayer MoS_2 nanoribbons. *J. Am. Chem. Soc*. **136**, 6269-6275 (2014).
3. Cheng, L. et al. Why two-dimensional semiconductors generally have low electron mobility. *Phys. Rev. Lett*. **125**, 177701 (2020).
